# Contrasting survival and physiological responses of sub-Arctic plant types to extreme winter warming and nitrogen

**DOI:** 10.1007/s00425-017-2813-6

**Published:** 2017-11-21

**Authors:** Stef Bokhorst, Laura Jaakola, Katja Karppinen, Guro K. Edvinsen, Hanne K. Mæhre, Jarle W. Bjerke

**Affiliations:** 1 Norwegian Institute for Nature Research (NINA), FRAM – High North Research Centre for Climate and the Environment, Langnes, PO Box 6606, 9296 Tromsø, Norway; 2 Department of Ecological Science, VU University Amsterdam, De Boelelaan 1085, 1081 HV Amsterdam, The Netherlands; 3 Climate Laboratory Holt, Department of Arctic and Marine Biology, UIT The Arctic University of Norway, 9037 Tromsø, Norway; 4 Norwegian Institute of Bioeconomy Research (NIBIO), PO Box 115, 1431 Ås, Norway; 5 Genetics and Physiology Unit, University of Oulu, PO Box 3000, FI-90014 Oulu, Finland; 6 Faculty of Biosciences, Fisheries and Economics, Norwegian College of Fishery Science, UIT The Arctic University of Norway, 9037 Tromsø, Norway

**Keywords:** C-repeat binding factor, Fatty acids, Frost, Grass, Multiple stresses, Shrub, Snow

## Abstract

**Evergreen plants are more vulnerable than grasses and birch to snow and temperature variability in the sub-Arctic.**

Most Arctic climate impact studies focus on single factors, such as summer warming, while ecosystems are exposed to changes in all seasons. Through a combination of field and laboratory manipulations, we compared physiological and growth responses of dominant sub-Arctic plant types to midwinter warming events (6 °C for 7 days) in combination with freezing, simulated snow thaw and nitrogen additions. We aimed to identify if different plant types showed consistent physiological, cellular, growth and mortality responses to these abiotic stressors. Evergreen dwarf shrubs and tree seedlings showed higher mortality (40–100%) following extreme winter warming events than *Betula pubescens* tree seedlings and grasses (0–27%). All species had growth reductions following exposure to − 20 °C, but not all species suffered from − 10 °C irrespective of other treatments. Winter warming followed by − 20 °C resulted in the greatest mortality and was strongest among evergreen plants. Snow removal reduced the biomass for most species and this was exacerbated by subsequent freezing. Nitrogen increased the growth of *B. pubescens* and grasses, but not the evergreens, and interaction effects with the warming, freezing and snow treatments were minor and few. Physiological activity during the winter warming and freezing treatments was inconsistent with growth and mortality rates across the plants types. However, changes in the membrane fatty acids were associated with reduced mortality of grasses. Sub-Arctic plant communities may become dominated by grasses and deciduous plants if winter snowpack diminishes and plants are exposed to greater temperature variability in the near future.

**Electronic supplementary material:**

The online version of this article (doi:10.1007/s00425-017-2813-6) contains supplementary material, which is available to authorized users.

## Introduction

The Arctic is experiencing more extreme weather events due to climate change, causing high mortality rates among species when events surpass survival thresholds (Post et al. 2009; Liston and Hiemstra 2011). Especially, winter is expected to experience more extreme events such as rain on snow, unseasonal warm periods, ground ice formation and loss of snow cover (Post et al. 2009; Vikhamar-Schuler et al. 2016). Abrupt changes in winter snow cover and depth following midwinter thaw events (e.g., from − 20 to + 5 °C in 24 h) affect plant survival as snow insulates against temperature extremes (Bokhorst et al. 2016). In addition, loss of snow cover during a midwinter melt can induce plant physiological activity, while the plants are generally dormant (Ögren 1996; Schaberg et al. 1996; Bokhorst et al. 2010). Evergreen plants seem to be more susceptible than deciduous plants and grasses to such events, but the cause of this is unclear (Bokhorst et al. 2009, 2011, 2015). Evergreen plants have more ‘alive’ green tissue aboveground and can potentially respond more strongly to abiotic cues making them more susceptible to damage, whereas deciduous plants have dormant buds which may take longer to develop and respond to midwinter warming events. Plant mortality can result from loss of frost hardiness due to physiological activity induced by the winter warming event (Strimbeck et al. 1995; Beck et al. 2004; Buchner and Neuner 2011; Pagter and Arora 2013) or increased freeze–thaw cycles and lower freezing temperature from the reduced snow cover (Lund and Livingston 1999). In addition, frost drought and leaf cuticle abrasion may result in tissue damage (Hadley and Smith 1986, 1989; Neuner et al. 1999). Identifying the drivers behind plant vulnerability allows for a better understanding of sub-Arctic vegetation changes, as the predictions of winter climate and snow conditions vary across circumpolar regions (Liston and Hiemstra 2011).

Basic physiological processes, such as respiration and the activity of the photosynthetic apparatus, slow down or stop altogether in response to colder winter temperature as a protection against freezing damage (Inouye 2000). This cold hardening slowly builds up during the autumn–winter transition under genetic control (Hughes and Dunn 1996; Thomashow 1999). The composition of fatty acids in the cell membrane plays an important role in the protective actions against frost damage (Steponkus 1984; Hughes and Dunn 1996), and many plants increase the unsaturated and longer fatty acid chains to limit membrane denaturation by freeze dehydration (Uemura et al. 1995; Uemura and Steponkus 1999; Dalmannsdóttir et al. 2001; Strimbeck et al. 2015). However, it is unclear if sub-Arctic plants can re-initiate these cold hardening processes following an abrupt midwinter warming event. Furthermore, extreme winter warming events occur against a background of gradually increasing temperature and anthropogenic disturbances such as nitrogen deposition.

Atmospheric nitrogen (N) deposition is a global threat to biodiversity and ecosystem function. Critically, even low-dose N deposition (5–10 kg N ha^−1^ year^−1^) may lead to eutrophication of the ecosystem and affect plant communities (Phoenix et al. 2012; Choudhary et al. 2016). In addition, many plants show changes in cell and physiological characteristics that are associated with drought and frost susceptibility when exposed to N deposition (Macgillivray et al. 1995; Carroll et al. 1999; Schaberg et al. 2002). As such, increased N inputs from agricultural practices or biomass burning reaching high latitudes (Forsius et al. 2010; Karlsson et al. 2013) could make sub-Arctic plants more vulnerable to the temperature variability of extreme winter warming events (Power et al. 1998).

To address these issues, we initiated a common garden experiment where the physiology (respiration rates, changes in fatty acid composition and gene expression of frost-resistant genes) of dominant sub-Arctic plant types (grasses, dwarf shrubs and trees) was monitored during simulations of extreme winter warming events in combination with N additions in sub-Arctic Norway. We expected that (1) evergreen plants would show larger growth reductions and mortality rates as compared to deciduous plants and grasses in response to extreme winter warming events because evergreens have more green tissue underneath the snow; (2) evergreen mortality would result from physiological activity in response to the winter warming period combined with a lack of physiological adaptations (changing fatty acid composition and gene expression); (3) freezing (− 10 and − 20 °C) following the extreme winter warming events will result in higher mortality rates irrespective of plant growth form, and that the loss of snow cover will exacerbate these effects; and (4) N addition will induce higher mortality when combined with extreme winter warming events. Identifying the strategies of various plant species and groups, such as evergreen dwarf shrubs, grasses and deciduous and evergreen trees, will provide predictive tools for modeling future sub-Arctic vegetation changes and carbon and nutrient cycling rates (Cornelissen et al. 2007; De Deyn et al. 2008).

## Materials and methods

### Extreme winter warming manipulation and simulation of nitrogen deposition

To determine the vulnerability of sub-Arctic plants to winter temperature variability, plants were collected from the field or grown from seeds during spring 2013 (see details below) and divided across three temperature treatments in the experimental garden at Holt (Tromsø, Norway, 69°40′N, 18°56′E). For the simulation of the extreme winter warming events and freezing, we removed plants from the experimental garden during January 2014 and exposed them to various temperature regimes under controlled conditions in climate chambers after which the plants were returned to the experimental garden for the remainder of winter (Fig. S1). The ambient climate conditions of the Tromsø area during the winter months of 2014 are presented in Table S1. A group of potted plants (*n* = 40 replicate potted plants per species) was left in the experimental garden as a control, while snow was removed from the remainder of the plants and divided across two dark climate chambers at 0.5 and 6.0 °C for a week (*n* = 120 replicate potted plants per species for each temperature). The 0.5 °C temperature was considered an ambient ‘treatment control’ (TC) of the subnivean temperature, while the 6.0 °C temperature represents the ‘extreme winter warming’ (WW) (Bokhorst et al. 2009). To quantify the impact of freezing on the survival and growth of the study plants following 3 days at TC or WW, we split the 120 replicate pots per species across three groups: one with exposure to − 10 °C, another with exposure to − 20 °C, and the third remained at the respective TC (0.5 °C) or WW (6.0 °C) (*n* = 40 per species for each treatment). The exposure of plants to − 10 or − 20 °C was done in dark climate chambers. The temperature in the climate chambers was gradually lowered from 0 to − 10 °C at 1 K h^−1^ and at 2 K h^−1^ to − 20 °C. After 2 days at the respective freezing, the plants were returned to their starting temperature (0.5 or 6.0 °C) for two more days. Following these treatments, all plants were returned to the field but only half of each treatment received a new snow cover; meaning that for every temperature treatment combination there was one insulated by snow (+ S), while the other was left exposed to variable ambient freezing conditions for the remainder of winter due to the lack of snow cover (− S). As an example of the full experimental design for the 0.5 °C treatments (TC) we now have: ‘TC + S’, ‘TC − S’, ‘TC exposed to − 10 °C + S’, ‘TC exposed to − 10 °C − S’, ‘TC exposed − 20 °C + S’ and ‘TC exposed − 20 °C − S’. The plants from the + S and − S pairs of each temperature treatment (e.g., ‘TC + S’ and ‘TC − S’) were placed in two adjacent rectangles (120 cm × 240 cm) 1 m apart with the potted plants in trays (60 cm × 30 cm) randomly placed within each rectangle.

To avoid crushing the plants with the manual build of a new snowpack, the plants were first covered with 20 cm of fine lightweight hoarfrost, collected from the surrounding snow surfaces, after which the snow level was brought back up to 80 cm with shoveled snow from the surrounding area. Snow was also removed from half of the plants of the control treatment (already split into two groups during summer 2013) to quantify the impact of snow removal on plant survival and growth when not exposed to extreme winter warming events (‘C + S’ and ‘C − S’). After the treatments, the plants were left untouched for the remainder of winter and spring until collected for harvesting during summer (mid-July) 2014. Soil surface temperature (0–1 cm depth) was measured at hourly intervals from autumn (2013) to spring (2014) by a temperature logger (I-Button, Maxim integrated, San Jose, CA, USA) in one potted plant for each treatment. From these data, we quantified the minimum temperature, the number of freezing events (freeze–thaw cycles) and spring thaw date.

To quantify the impact of nitrogen (N) addition in combination with extreme winter warming effects, half of each experimental treatment received additional N (5 kg N m^−2^ year^−1^) during the growing season (2013) before the extreme winter warming events. N was applied by watering the plants twice during the growing season (30 July and 15 August). The N treatments received in total 2 L of an ammonium nitrate (NH_4_–NO_3_) solution, while the non-nitrogen plants received an equal amount of tap water. The total amount of water added was less than 1% of the mean annual precipitation falling in Tromsø (1031 mm). There were ten replicate plants per species for each temperature–nitrogen treatment.

### Study species

As we expected to find contrasting vulnerability between plant species and plant growth forms to extreme winter warming events (Bokhorst et al. 2015), we aimed to compare the differences between dominant sub-Arctic plant species and plant functional types. As representatives of evergreen plants, we used the dwarf shrubs *Empetrum nigrum* and *Vaccinium vitis*-*idaea*, tree seedlings of *Picea abies* and *Pinus sylvestris* (grown from seeds during the summer preceding the winter treatments) and the rosette plant *Pyrola grandiflora* ssp. *norvegica*. We used seedlings of the deciduous tree *Betula pubescens* and the three grasses *Poa alpina*, *Phleum alpinum* and *Festuca rubra* ssp. *richardsonii* which retain some overwintering leaves (all grown from seeds during the summer preceding the winter treatments). The grasses are included in the deciduous group in this study as their main green biomass has to regrow in spring. *P. alpina*, *P. alpinum* and *F. rubra* are a dominant component of many sub-Arctic ecosystems and their overwintering leaves allow for physiological measurements during winter which would be limited if all leaves senesce during autumn. From here on, genus names will be used to identify the different species. The grass and tree species were all grown from seeds in greenhouses at the climate laboratory Holt in Tromsø and transferred to individual pots (5 cm × 5 cm × 5 cm) with potting soil (spring 2013). A total of 1680 potted individuals (*n* = 280 for each tree and grass species) were used in the experiment with 20 individuals (pots) per species used as replicates for each treatment of which half received additional N (*n* = 10 with additional N and *n* = 10 without N).

As the dwarf shrubs are very slow growing, we collected complete soil–vegetation mats (60 cm × 30 cm and approximately 7–9 cm soil) dominated by *Empetrum* and *Vaccinium* from a Scots pine forest in the interior of Troms County (69°23′N, 20°16′E), with similar elevation and exposure to the experimental garden during April 2013. The soil–vegetation mats were kept in plastic trays (60 cm × 30 cm) filled with clean river sand to allow root growth. *Pyrola* individuals (including roots and peat) were collected by hand from a fen in the same area and planted within the soil–vegetation mats dominated by *Empetrum* and *Vaccinium*. Due to the space limitations in the climate chambers for the winter treatments, we used ten replicate trays with *Empetrum* and *Vaccinium* and *Pyrola* for each treatment (*n* = 5 with additional N and *n* = 5 without N).

### Plant survival/mortality

During mid-July 2014, we calculated the relative population mortality rates for each species assuming that necrosis of the aboveground plant parts (complete browning) was indicative of complete dieback of plant individuals. As we lack individual plants for the mat-forming dwarf shrubs *Empetrum* and *Vaccinium*, we calculated % mortality from the relative mass of dead shoots compared to total standing aboveground biomass (including both alive and dead shoots based on oven dry mass) in each soil–vegetation mat during mid-July. For the grasses and trees, we quantified individual plant total biomass (shoots and roots) after drying (70 °C for 48 h).

### Winter physiological activity

During the winter manipulations, we collected the leaf tips of the green overwintering tissue of the grasses, intact leaves of *Vaccinium* and *Picea* and shoot tips of *Empetrum*, except from *Betula* and *Pinus* as leaves were not available for *Betula* and there were not enough leaves of *Pinus*. These samples were analyzed to quantify the winter physiological activity of the plants (respiration rates and potential activity of PSII; Fv/Fm), potential cell membrane damage through electrolyte leakage, fatty acid composition of cell membranes and expression of the *C*-*repeat binding factor* (*CBF*) gene involved in frost resistance. Samples were collected after 3 days at the respective acclimation temperature (TC and WW) and 24 h following the freezing treatments.

Leaf respiration was measured in complete darkness using a leaf gas exchange system (GFS-3000, Walz GmbH, Effeltrich, Germany) with the measuring head set at 0.5 or 6 °C, depending on the acclimation temperature (TC or WW) of the plants, and 7000 ppm H_2_O, with a 380-ppm base level of CO_2_. We used Walz’ cuvette 3010-V80, which is specifically designed for small, loose samples. For small individual leaves or tissue, we turned the cuvette upside down, so that the leaves were on top of the ventilated lid and thereby closer to the light source and emitting a stronger fluorescence signal. Leaf samples were collected from individual plants after 3 days at their acclimation temperature (0.5 or 6 °C) and following freezing. Tissue samples were measured within 5 min of collection to obtain a representative estimate of physiological activity between treatments. We used the mean respiration rates of nine measurements taken at 15 s intervals for each sample (*n* = 5 per species for each treatment). Fv/Fm was quantified for each leaf/tissue sample at the end of the gas exchange measurements. As the samples were not exposed to any light sources during sampling, we assume that the leaves were properly dark adapted for fluorescence measurements.

The electrolyte leakage method was used to assess the potential damage of cell membranes due to freezing (Caporn et al. 1994). Intact leaves, although grass leaves were cut, were sampled from each species before and after freezing (*n* = 10 per species for each treatment). Samples were placed in a plastic tube with 30 mL distilled water, shaken briefly and stored at room temperature for 6 h. The conductivity of the solution was measured with a conductivity meter (HI 9835; Hanna Instruments, Leighton Buzzard, UK) after this period to quantify the initial electrolyte loss, although some authors use longer sample storage periods to increase electrolyte leakage values (Murray et al. 1989; Kathke and Bruelheide 2011). The sealed tubes with plant samples were then autoclaved (120 °C for 4 h) to completely destroy the integrity of the cell membranes and release all electrolytes to acquire the maximum conductivity of each sample. The ratio of the initial to the maximum conductivity was calculated and compared across the treatments with high values indicative of a higher proportion of cell membrane damage.

To quantify changes in the fatty acid composition of the membranes as a result of the warming and freezing treatments, we collected leaf samples (*n* = 5), while plants had been acclimated to TC (0.5 °C) or WW (6.0 °C) for 3 days and following the freezing (− 10 and − 20 °C) treatments. All samples were immediately frozen at − 20 °C, freeze dried, ground and analyzed for fatty acids following the direct methylation procedure (Browse et al. 1986). Samples of 5–20 mg were dissolved in 1 mL methanolic hydrochloric acid (HCl) (1 M) and an internal standard (heptadecanoic acid, C17:0) was added to a glass tube. The solution was heated to 80–100 °C for 1 h, and after cooling 0.4 mL hexane and 1 mL of 0.9% sodium chloride (NaCl) were added to each sample. The fatty acid methyl esters (FAMES) were extracted into the hexane phase by vigorous shaking on a benchtop shaker (10 s). The tubes were centrifuged for 10 min (at 212*g*) to separate the phases completely, and a sample was then taken directly from the hexane phase. Samples were stored at − 20 °C until gas chromatography (GC) analyses, according to Mæhre et al. (2013). The instrument used was an Agilent 6890N equipped with a flame ionization detector (FID) (Agilent Technologies Inc., Santa Clara, CA, USA) and a CP7419 capillary column (50 m × 250 µm × 0.25 µm nominal, Varian Inc., Middelburg, the Netherlands). The fatty acids were identified by comparing against the commercial fatty acid standards PUFA 1, 2 and 3 (Sigma-Aldrich, St. Louis, MO, USA) and the GLC standards 80, 411 and 412 (NuChec Prep. Inc., Elysian, MN, USA). The amount of each fatty acid was calculated by comparing the peak area with the known amount of an internal standard (C17:0).

To quantify the expression of the frost-resistant *CBF* gene, plant tissues were sampled before, during and after the freezing treatments (− 10 and − 20 °C). The samples were immediately placed at − 80 °C until used for RNA extraction. Total RNA was isolated from the tissues according to Jaakola et al. (2001) with the exception that the phenol–chloroform extraction was substituted with the RNA purification protocol of E.Z.N.A. Total RNA Kit I (Omega Bio-Tek, Norcross, GA, USA) after treatment with DNase I (Sigma-Aldrich). The cDNA was synthesized from total RNA using the SuperScript VILO cDNA Synthesis Kit (Invitrogen, Carlsbad, CA, USA) according to the manufacturer’s instructions. Sequences of the *Empetrum CBF* gene were isolated by PCR with degenerate oligonucleotide primers 5′-GATGGCTGCTAGGGCTCAYGAYGTNGC-3′ (forward) and 5′-GGAGGCAGCAGCATTCCYTCNGCCAT-3′ (reverse) that were designed using the CODEHOP software (Rose et al. 2003) based on conserved regions found in previously isolated dicot *CBF* proteins. *Empetrum* DNA extracted with E.Z.N.A. HP Plant DNA Mini Kit (Omega Bio-Tek) was utilized for amplification of *CBF* fragments by using Phusion DNA polymerase (ThermoFisher Scientific Inc., Waltham, MA, USA) under standard PCR conditions with annealing temperature of 54 °C. The amplified PCR product was gel-purified with E.Z.N.A. Gel Extraction Kit (Omega Bio-Tek) and ligated into a pJET cloning vector using CloneJET PCR Cloning Kit (ThermoFisher). Sequencing was performed using a BigDye Terminator Cycle Sequencing Kit (Applied Biosystems, Foster City, CA, USA) and sequenced at the Sanger facility in Tromsø. The nucleotide sequence of *Empetrum CBF* is deposited in GenBank under Accession number KX618729. The quantitative reverse transcription PCR (qRT-PCR) analysis to determine transcript abundance of *Empetrum CBF* and *Vaccinium CBF* (GenBank Accession no. JN866912; Oakenfull et al. 2013) was performed with a MiniOpticon instrument (Bio-Rad, Hercules, CA, USA) using SsoFast™ EvaGreen Supermix (Bio-Rad). The qRT-PCR conditions were an initial incubation at 95°C for 10 min followed by 45 cycles of 95°C for 10 s, 60°C for 20 s, and 72°C for 10 s. For the relative quantification of PCR products, 18S ribosomal RNA was used as a reference gene. The gene-specific primer sequences used for the qRT-PCR analysis are shown in Supplementary Table S2. The results were calculated using the calibrator-normalized PCR efficiency-corrected method using the CFX Manager software 2.0 (Bio-Rad). The amplification of only one product in qRT-PCR was confirmed by a melting curve analysis. Quantifying the expression of *CBF* genes was only successful for *Empetrum* and *Vaccinium*. Thus, we were unable to do the planned comparison of evergreen and deciduous plant responses.

### Statistical analyses

To compare the impact of treatments (C, TC and WW), extremes (− 10 and − 20 °C), N addition and snow removal on plant survival, we used a Chi-square test with the following null hypothesis: ‘There is no difference in the number of dead and alive plants between the treatment comparisons’. We used a factorial ANOVA to compare the impact of winter warming (TC: 0.5 °C vs WW: 6.0 °C), temperature extremes (0.5, 6.0, − 10 and − 20 °C) and N addition (0 vs. 5 kg m^−2^ year^−1^) on plant biomass, physiological variables and the concentration of fatty acids. As plant biomass was quantified during the following growing season, we also included the impact of snow removal in the factorial ANOVA. To summarize the large number of fatty acids into fewer variables, we performed a principal component analysis (PCA) on the proportional data for each of the identified fatty acids. We compared the impact of winter warming, temperature extremes and N addition on the scores of PC1 and PC2. There were few significant interaction terms, and we therefore only show the main effect responses in the table and figures. Where relevant, the interaction terms are mentioned in “Results”. In all cases, homogeneity of variance was tested with a Levene’s test of equality, and log transformation was applied when necessary. All statistical analyses were carried out using R 3.3.0 (R Core Team 2015).

## Results

### Impact of temperature treatments and snow removal on the plant environment

**Fig. 1:**
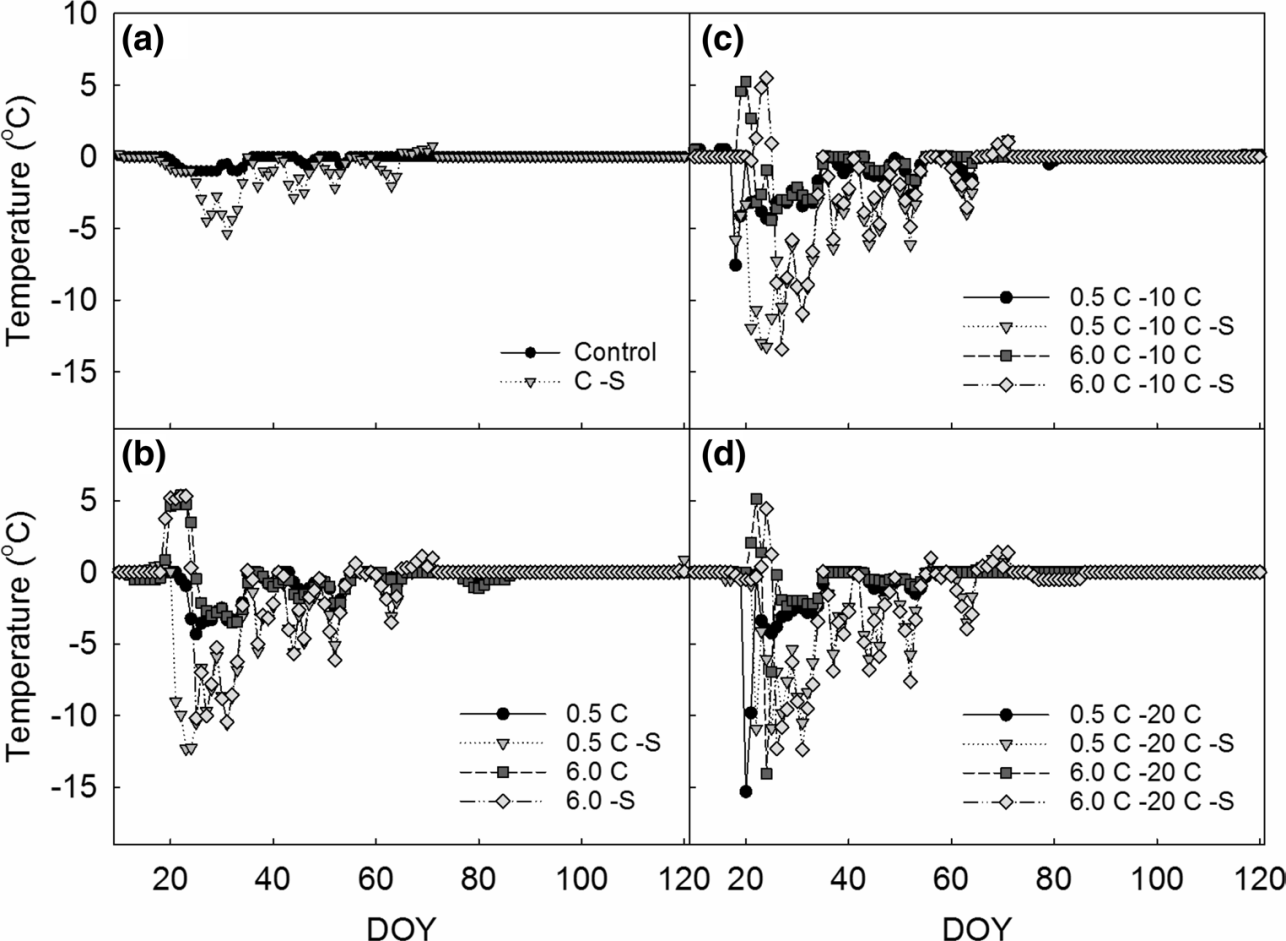
Daily mean soil surface temperature during and following the extreme winter warming and freezing experiments. **a** Soil surface temperature in control plots with and without snow (− S). **b** Temperature in the ‘Treatment Control’ (0.5 °C) and ‘WW’ (6 °C) treatment with and without snow. **c** Soil surface temperature of the potted plants during and following exposure to − 10 °C (for TC and WW) with and without snow. **d** Soil surface temperature of the potted plants during and following exposure to − 20 °C (for TC and WW) with and without snow. Days of the year (DOY) from 1 January until 1 May are shown on the horizontal axes

Snow removal lowered the minimum soil surface temperature in the control plots to − 5 °C during the remainder of winter, while temperature did not fall below − 1 °C when the snow layer was intact (Fig. 1a). Minimum temperature was lower (− 12 °C) in some of the other snow removal treatments due to a few days with colder frost during which both control plots were still covered by snow (Fig. 1b–d). Minimum soil temperature measured in the − 10 °C treatment was − 7 °C (Fig. 1c) and − 15 °C in the − 20 °C treatment (Fig. 1d). There was no difference in the number of freeze–thaw cycles between snow-covered (4 ± 1) and snow removal plots (5 ± 1). There was no significant difference between treatments in the date of spring thaw, i.e., the date preceded by three consecutive days with daily mean temperature above 0 °C (day of year 128).

### Impact of winter temperature variability on population mortality

**Fig. 2:**
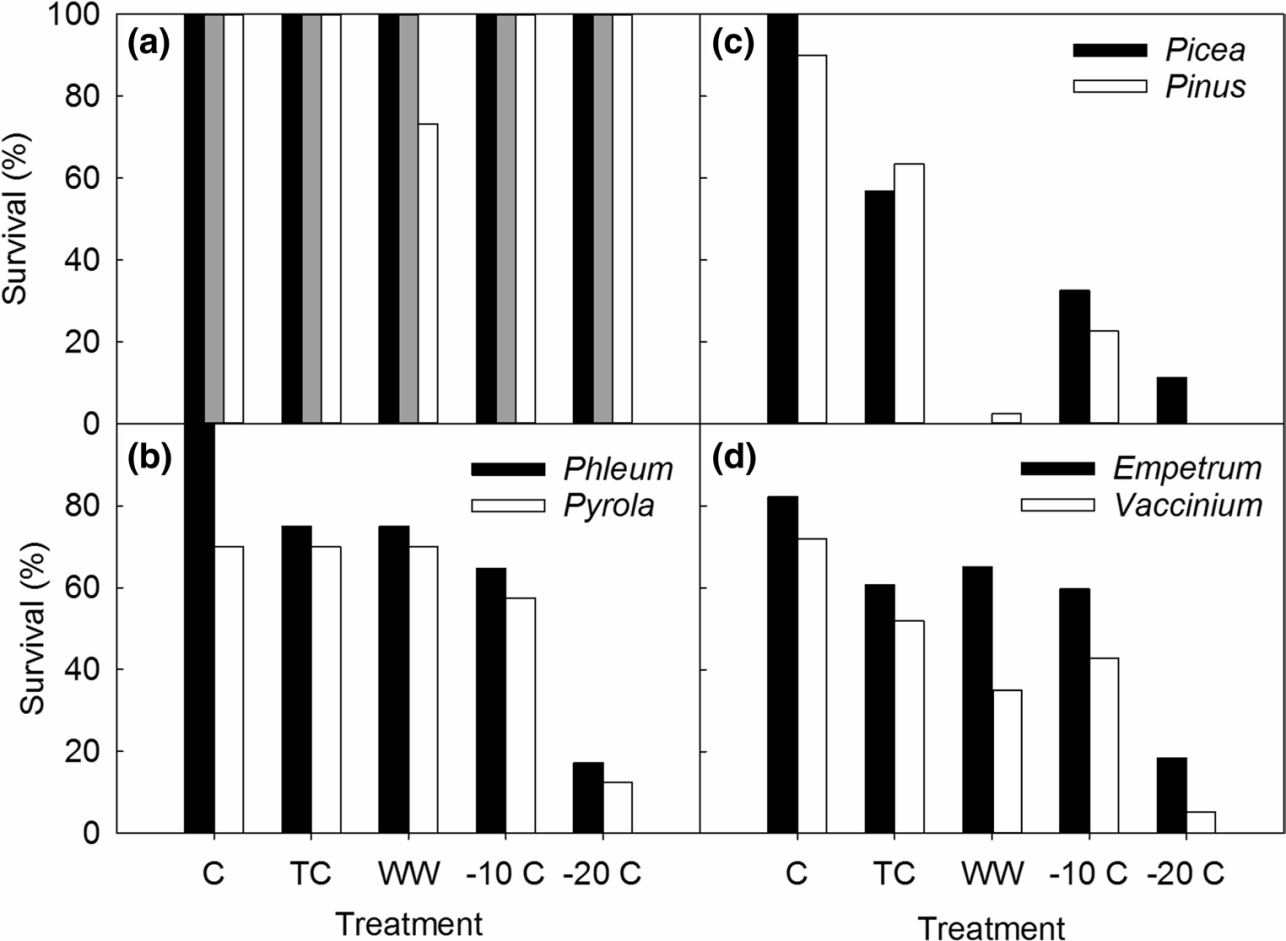
Survival of sub-Arctic plants following simulations of extreme short winter warming events. The treatments included a true control (C), a treatment control (TC at 0.5 °C), a winter warming treatment (WW at 6 °C) and freezing exposure to − 10 and − 20 °C. **a** All *Betula* (black) and *Festuca* (gray) survived, while 73% of *Poa* (white) plants survived when exposed to winter warming. **b** Survival of the grass *Phleum* and rosette plant *Pyrola*. **c** Survival of evergreen tree seedlings (*Pinus* and *Picea*). **d** Survival of evergreen dwarf shrubs (*Empetrum* and *Vaccinium*)

*Betula* and *Festuca* plants survived the winter treatments (Fig. 2a). *Poa* mortality was limited to WW (27%): specifically, 100% mortality following the combination of: WW, − 20 °C, + N and − S (Fig. 2a). *Phleum* survived in the control plots, but there was 25% mortality in TC and WW. In addition, freezing to − 10 and − 20 °C resulted in 35 and 83% mortality, respectively (Fig. 2b). Snow removal resulted in 51% *Phleum* mortality, compared to 30% with snow cover, but WW and freezing increased mortality to 80% (− 10 °C) and 100% (− 20 °C). Overall, there was no effect of N on mortality for any of the deciduous species (birch and grasses).

Most of the evergreen plants sustained considerable mortality following the WW and freezing, although there were differences between species. *Pyrola* mortality was 30% in the control, TC and WW, while freezing to − 10 and − 20 °C increased mortality to 43 and 88%, respectively (Fig. 2b). There was no effect of N or − S on *Pyrola* mortality. *Picea* mortality was 0, 43 and 100% in C, TC and WW, respectively, and 68 and 89% following the − 10 and − 20 °C freezing (Fig. 2c). There were no consistent effects of N or − S on mortality. 98% of *Pinus s*eedlings died in WW, 53% died in TC, while 10% died in the control. *Pinus* seedlings did not survive the − 20 °C and only 22% survived the − 10 °C freezing (Fig. 2c). *Pinus* exposed to ‘TC − 10 °C + N’ did not survive, while those without N did. Mortality, based on biomass, of *Empetrum* and *Vaccinium* did not differ between TC, WW and − 10 °C, but was highest after − 20 °C (Fig. 2d). There was no main effect of – S on *Empetrum* mortality, except following the most extreme event (WW − 20 °C) when mortality was 67% with snow and 97% without snow. Snow removal increased the mortality of *Vaccinium* to 67% compared to 60% mortality when plants were covered by snow.

### Impact of extreme events on subsequent biomass production of surviving plants

**Fig. 3:**
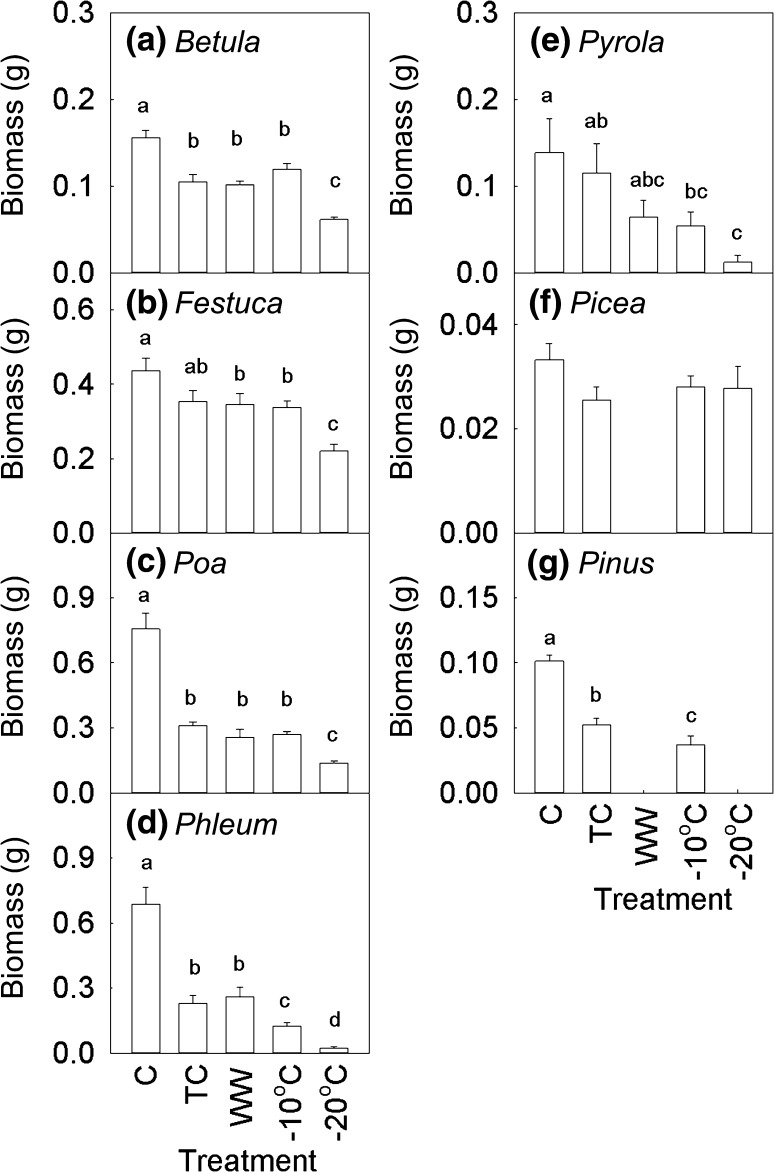
Impact of extreme short winter warming events and freezing on total plant biomass. The treatments included a true control (C), a treatment control (TC at 0.5 °C), a winter warming treatment (WW at 6 °C) and freezing exposure to − 10 and − 20 °C. Biomass values are obtained from plants that survived (see Fig. 2). Bars with different letters are significantly different (Tukey’s HSD *P* < 0.05) (see Table 1 for ANOVA results). Bars are means of *n* = 40–120, as the number of plants that survived varied across species. Error bars are 1 SE

All plants, except *Festuca* and *Picea*, experienced decreased biomass following the transferral from the subnivean environment to the climate chambers compared to the control plots that were left undisturbed in their subnivean environment the entire winter (Fig. 3). *Betula*, *Festuca* and *Poa* showed reduced biomass following − 20 °C, while no biomass differences were found between TC, WW and − 10 °C (Fig. 3a–c). The biomass of *Festuca* and *Poa* was 43 and 50% lower in ‘WW – 20 °C’ than in ‘TC – 20 °C’. *Phleum* showed an intermediate response in that plants exposed to − 10 °C and had lower biomass compared to TC and WW, while − 20 °C was even lower (Fig. 3d). The biomass of the evergreens *Pyrola* and *Pinus* gradually decreased with temperature extremes (Fig. 3e, g), while *Picea* biomass was very low and did not differ between any of the treatments (Fig. 3f).

**Fig. 4:**
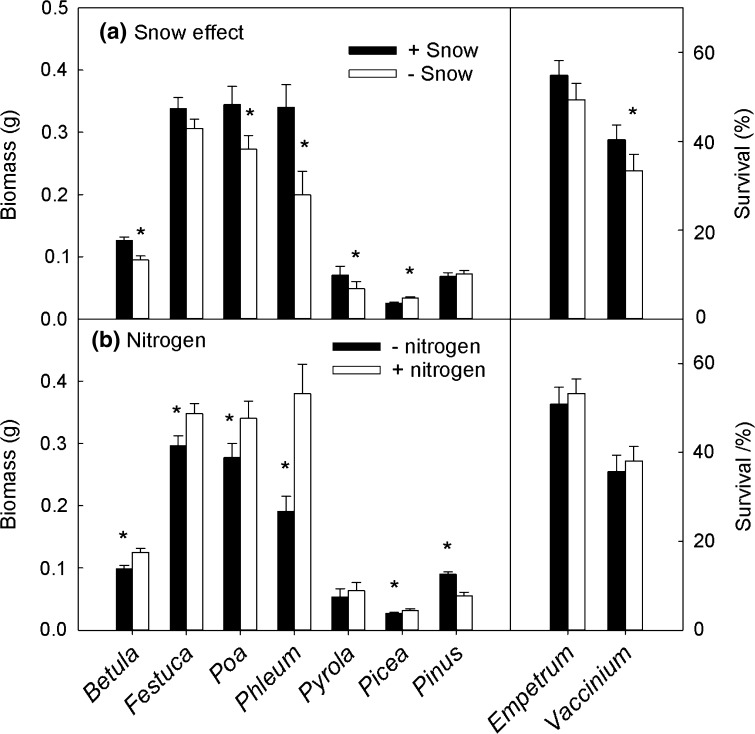
Impact of snow removal (**a**) and nitrogen addition (**b**) on various sub-Arctic plant biomass and survival. Note that for the evergreen dwarf shrubs *Empetrum nigrum* and *Vaccinium vitis*-*idaea* there was no individual plant biomass data; hence, the % survival for the whole soil–vegetation mat is presented. Asterisks indicate significant (*P* < 0.05) differences between treatments (see Table 2 for ANOVA results). Bars are means of *n* between 45 and 157, as sample sizes varied across species. Error bars are 1 SE

Snow removal led to the reduced biomass of *Betula* (25%), *Poa* (21%) and *Phleum* (41%), but not consistently for *Festuca* (Fig. 4a). *Festuca*, *Poa* and *Phleum* biomass was 47, 58 and 76%, respectively, lower following WW without snow compared to WW with snow cover (Treatment × Snow: *P* < 0.05). Snow removal increased *Picea* biomass by 30% (and this effect was highest in the C plots (73%), while for *Pyrola* the biomass was reduced by 27%, although this effect was primarily restricted to the TC and WW treatments (with 9 and 3 times less biomass following snow removal). *Pinus* biomass was not affected by snow removal.

Nitrogen addition led to increased biomass of birch and grasses (Fig. 4b). However, this effect disappeared for *Poa* in the WW treatment (Treatment × N: *F*_2,256_ = 4.6, *P* < 0.05). *Picea* biomass increased by 18% in response to N addition, although this effect was limited to the control plants. *Pinus* biomass was reduced by 39% in response to N addition. *Poa* was the only species with a significant snow × nitrogen interaction (N × S: *F*_1,256_ = 6.8, *P* < 0.01) resulting in a 44% higher biomass when provided a snow cover and N. This N × S effect was significant for C and WW, but not for TC.

### Ecophysiological responses during winter

**Fig. 5:**
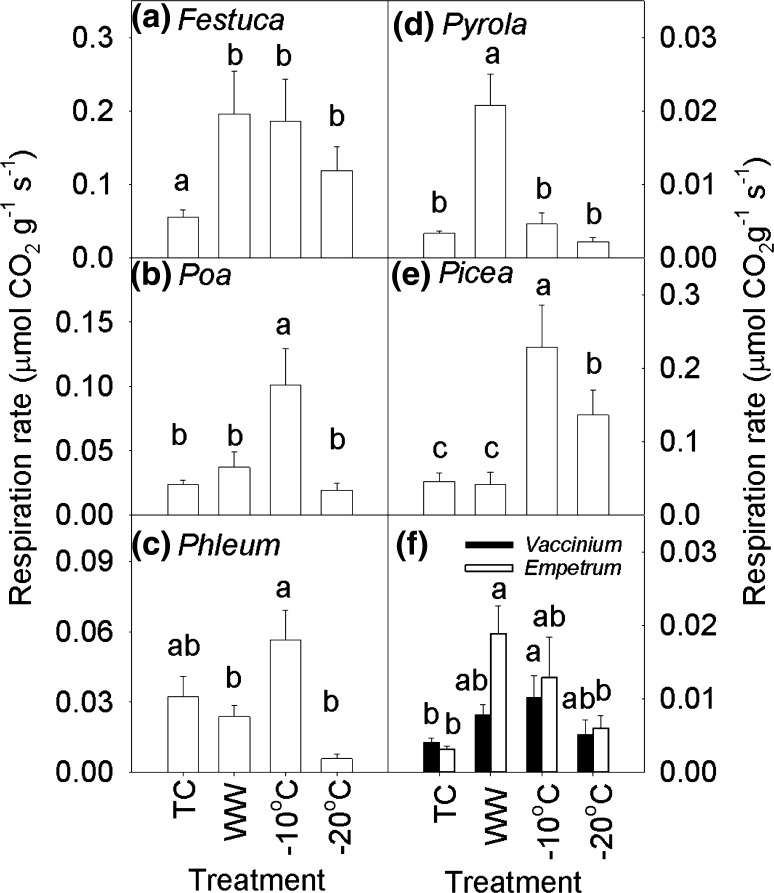
Winter respiration rates of sub-Arctic plants exposed to extreme short winter warming events and freezing. The treatments included a treatment control (TC at 0.5 °C), a winter warming treatment (WW at 6 °C) and freezing exposure to − 10 and − 20 °C. Bars with different letters are significantly different from one another (Tukey HSD *P* < 0.05). Bars are means of *n* = 20. Error bars are 1 SE

Ecophysiological responses to winter temperature variability varied greatly between and within plant functional groups (Table 2). *Festuca* respiration rates more than doubled compared to TC, while *Poa* and *Phleum* showed increased respiration only following − 10 °C freezing (Fig. 5). Respiration rates of *Pyrola* and *Empetrum* were six times higher in WW compared to TC, while the strongest responses were measured following − 10 °C freezing compared to TC for *Picea* (5 times higher) and *Vaccinium* (2 times higher). Fv/Fm of *Festuca*, *Phleum* and *Vaccinium* decreased (38, 26 and 34%, respectively) following freezing, while Fv/Fm of the other species was not affected (Fig. S2). Electrolyte leakage in *Poa*, *Phleum*, *Pyrola* and *Vaccinium* increased (71, 76, 64 and 110%, respectively) following freezing, while it was not affected in *Festuca* and *Empetrum* (Fig. S3). *Picea* had the highest electrolyte leakage in TC and the lowest following the − 20 °C freezing (Fig. S3). *Phleum* responded more strongly to − 20 °C following 0.5 °C (66% loss of electrolytes) than to − 20 °C following 6.0 °C (44% loss). N addition reduced the electrolyte leakage of *Festuca* (without N: 44% loss; with N 38%). N addition increased electrolyte leakage in *Poa* following the − 20 °C freezing in combination with WW (94% loss) compared to the same treatment without N (61% loss).

**Table 1:** Chi-squared and ANOVA statistics of plant survival and biomass of deciduous and evergreen species exposed to extreme winter warming simulations [Control, Treatment Control (0.5 °C) and Winter Warming], freezing (− 10 and − 20 °C), snow removal (+ Snow vs. − Snow) and nitrogen additions (0 vs. 5 kg N m^−2^ year^−1^)

	Mortality (Chi squared)				Biomass (*F* values)			
	Warming	Freezing	Nitrogen	Snow	Warming	Freezing	Nitrogen	Snow
*Betula*	No mortality				11.9***	17.2***	20.5***	16.6***
*Festuca*	No mortality				13.5***	12.0***	6.2*	2.1
*Poa*	16.3***	67.8***	9.0**	9.6**	155.7***	17.9***	14.6***	12.1***
*Phleum*	39.0***	97.2***	0.1	12.3***	77.8***	7.4***	9.4**	28.3***
*Pyrola*	4.1	31.3***	0.1	0.1	4.2*	0.8	0.0	5.2*
*Picea*	141.5***	117.4***	0.1	0.1	3.5	0.2	4.1*	5.1*
*Pinus*	122.5***	145.2***	2.6	0.1	106.8***	40.3***	65.8***	0.2
					% Dead biomass			
*Empetrum*					35.1***	53.5***	0.7	3.5
*Vaccinium*					57.1***	39.9***	0.6	5.4*

With the Chi-square analysis, we tested the following null hypothesis: ‘There is no difference in the number of dead and alive plants between the treatment comparisons’, and lack of significance confirms this hypothesis. Note that there was no mortality among *Betula* and *Festuca* in response to any of the treatments. Only main factors are shown for the ANOVA as there were few consistent significant interactions terms. Relevant interaction terms are mentioned in the results text. * *P* < 0.05, ** *P* < 0.01, *** *P* < 0.001

**Table 2:** ANOVA statistics (*F* values) of plant respiration, potential activity of photosystem II (Fv/Fm), electrolyte leakage and changes in fatty acid composition of dominant sub-Arctic plant types exposed to extreme winter warming simulations

	*Festuca*	*Poa*	*Phleum*	*Pyrola*	*Picea*	*Vaccinium*	*Empetrum*
Respiration rate (μmol CO_2_ g^−1^ s^−1^)							
Warming treatment	12.8**	18.8***	4.8*	1.2	12.4**	24.0***	0.8
Extreme temperature	7.9***	18.3***	6.9***	35.5***	17.9***	10.4***	4.3**
Nitrogen addition	0.8	2.1	1.1	2.9	1.5	0.4	0.3
Fv/Fm							
Warming treatment	0.7	3.3	3.7	2.4	2.0	0.9	1.4
Extreme temperature	4.4**	2.3	12.2***	2.5	0.6	4.2**	0.5
Nitrogen addition	0.5	0.3	0.6	0.2	2.0	0.1	2.3
Electrolyte leakage (%)							
Warming treatment	0.3	3.3	0.0	3.2	21.9***	3.2	0.0
Extreme temperature	2.5	22.3***	9.3***	5.1**	17.4***	4.3**	1.8
Nitrogen addition	5.8*	0.3	2.4	2.5	0.0	0.4	0.0
Membrane fatty acids							
PC1							
Warming treatment	0.0	10.1**	9.5***	0.4	0.5	3.2	0.2
Extreme temperature	4.0**	7.1***	2.5	3.7*	2.8	2.1	1.1
Nitrogen addition	1.1	0.1	0.1	0.4	0.1	1.6	0.0
PC2							
Warming treatment	2.7	1.5	1.7	–	–	1.2	0.3
Extreme temperature	2.1	1.2	1.1	–	–	1.5	1.7
Nitrogen addition	0.5	0.1	1.6	–	–	4.6*	1.0

Winter warming treatments: TC: 0.5 °C, WW: 6.0 °C; extreme temperature treatments: 0.5, 6.0, − 10 and − 20 °C; nitrogen addition treatments: 0 or 5 kg N m^2^. * *P* < 0.05, ** *P* < 0.01, *** *P* < 0.001. Note that the snow removal on these variables could not be tested as the snow removal treatment took place after the warming and freezing treatments in the climate chambers

### Impact on membrane fatty acid composition

The detected fatty acid composition differed greatly between plant species, which limited direct comparisons of specific fatty acids between study species. However, changes in fatty acid composition in response to the treatments could be quantified. The PCA shows that grasses and *Pyrola* responded to the treatments by changing the composition of fatty acids (Table 2, Fig. S4). N addition hardly affected the plants and only the evergreen *Vaccinium* showed minor significant effects along PC 2 (Table 2). This change in *Vaccinium* was caused by a lower proportion (*F*_1,112_ = 4.3, *P* = 0.040) of c18:1 n-9 (N added: 23%; without N: 25%).

Fatty acid composition differed between the WW and − 20 °C treatments compared to TC in *Festuca* and *Poa*, and the − 10 and − 20 °C freezing differed from TC in *Phleum* (Table 3). The changing composition in *Festuca* was driven by significant increases of c8:0 and decreases of c10:0, c12:0, c14:0, c14:1 and c18:2 n-6. Treatment differences for *Poa* included increases in the proportion of c11:0, c15:0, c18:0, c18:2 n-6, c22:6 n-3 and an unidentified fatty acid and a decline in c18:3 n-6 for the − 20 °C freezing as compared to TC. Treatment differences for *Phleum* included decreases in c18:2 n-6, c18:3 n-3 and c22:6 n-3 in the − 20 °C freezing compared to TC, and c16:1 n-7 decrease in WW compared to TC. For *Pyrola*, we observed a higher proportion of c16:0 (14%) in the − 20 °C freezing compared to TC (11%), a lower proportion of c18:3 n-6 in the − 10 °C (43%) and − 20 °C (46%) freezing compared to TC (55%) and a higher proportion of c20:2 following the − 10 °C freezing (29%) compared to TC (17%).

**Table 3:** Differences in fatty acid proportions for three sub-Arctic grass species in response to extreme short winter warming events and freezing

Fatty acids	Treatments			
	TC	WW	− 10 °C	− 20 °C
*Festuca*				
c8:0	0.12 (0.01)a	0.24 (0.03)b	0.20 (0.04)ab	0.29 (0.04)b
c10:0	0.04 (0.01)a	0.02 (0)b	0.03 (0)ab	0.02 (0)b
c12:0	0.03 (0.01)a	0.01 (0)b	0.03 (0.01)a	0.01 (0)b
c14:0	0.04 (0.01)a	0.02 (0.01)b	0.04 (0.01)a	0.01 (0)b
c14:1	0.21 (0.03)a	0.11 (0.02)b	0.15 (0.02)ab	0.12 (0.02)b
c18:2n-6	0.001 (0)a	0.00 (0)b	0.001 (0)a	0.00 (0)b
*Poa*				
c11:0	0.00 (0)a	0.00 (0)ab	0.01 (0)b	0.01 (0)b
c15:0	0.01 (0)a	0.01 (0)ab	0.01 (0)ab	0.01 (0)b
c18:0	0.01 (0)a	0.01 (0)ab	0.02 (0)ab	0.02 (0)b
c18:2n-6	0.02 (0.01)a	0.03 (0.01)ab	0.06 (0.02)b	0.07 (0.01)b
c18:3n-6	0.17 (0.01)a	0.13 (0.01)ab	0.16 (0.01)ab	0.12 (0.01)b
c20:n-9	0.38 (0.03)a	0.32 (0.03)ab	0.35 (0.03)ab	0.27 (0.02)b
c22:6n-3	0.00 (0)a	0.02 (0.01)ab	0.03 (0.01)ab	0.04 (0.01)b
*Phleum*				
c16:1n-7	0.02 (0)a	0.01 (0)b	0.02 (0)ab	0.02 (0)ab
c18:2n-6	0.16 (0)a	0.13 (0.01)ab	0.15 (0.01)ab	0.13 (0.01)b
c18:3n-3	0.48 (0.02)a	0.43 (0.03)ab	0.35 (0.03)bc	0.32 (0.02)c
c22:6n-3	0.03 (0)a	0.03 (0.01)ab	0.04 (0.01)ab	0.05 (0.01)b

The treatments included a treatment control (TC at 0.5 °C), a winter warming treatment (WW at 6 °C) and freezing exposure to − 10 and − 20 °C. Note that only the fatty acids with significant proportional differences between treatments are shown. Values are means of *n* = 20–40 replicates as sample size varied across species and treatments. SEs are shown between brackets. Values with different letters between treatments indicate significant (*P* < 0.05) differences

### *CBF* expression

Expression of the *CBF* gene was greatly increased (30 to 100 times) following the − 10 and − 20 °C freezing for both *Empetrum* and *Vaccinium* (Fig. S5). There were no major differences between the TC and WW and no impact of N addition on the expression of *CBF* (data not shown).

## Discussion

This study was initiated to test if there are consistent drivers and responses between dominant functional plant types (evergreens, grasses and deciduous) in the sub-Arctic in response to extreme winter warming events against a background of increased nitrogen deposition. Evergreen dwarf shrubs and trees turned out to be more vulnerable to extreme winter warming events than grasses and a deciduous tree, confirming our first hypotheses and earlier assumptions following field simulations of extreme events and observations in nature following such events (Bokhorst et al. 2009, 2015). Furthermore, we wanted to test if there were consistent physiological responses behind the growth and mortality responses to such extreme events, but this was clearly not the case. However, the measured responses may have been underestimated in relation to what happens during extreme winter warming events in nature due to the partly artificial nature of the experiment. This aspect was revealed by the reduced plant biomass in the treatment control (TC) compared to control (C) for some of the study species. As such, the transferral to the climate chambers already had an impact on plant growth in the following season. These responses highlight the importance of events outside the growing season for plant growth rates during summer (Bokhorst et al. 2012).

Plant respiration and Fv/Fm did not show consistent responses between or among plant growth forms to the warming events, thereby not supporting the hypothesis that winter physiological activity is a primer for mortality. Winter plant physiological activity is potentially dangerous due to the risk of cell damage and freezing mortality (Schaberg 2000; Beck et al. 2004). However, our results indicate that there was no link between increased (*Festuca*, *Pyrola* and *Empetrum*) or very low (*Poa*, *Phleum*, *Picea* and *Vaccinium*) physiological activity and electrolyte leakage or mortality in response to extreme winter warming events. The winter physiology of *Picea* was poor irrespective of winter treatments and may be a factor behind the overall low summer biomass values, although there were consistent mortality responses to WW and freezing. Changes in fatty acids coincided with reduced mortality and growth limitation, suggesting that this physiological adaptation is an important strategy linked to coping with extreme winter temperature variability. Surprisingly, these changes could still occur despite plants being active for only a few days during the middle of winter. However, there were large differences between the grass species with *Poa* and *Phleum* showing increases of some of the longer unsaturated fatty acids (c22:6 n-3 docosahexaenoic acid) as expected (Senser and Beck 1982), while showing opposite responses for linoleic acid (c18:2 n-6) which is known to be associated with frost resistance in white clover (Dalmannsdóttir et al. 2001). *Festuca* showed increases and decreases of short saturated fatty acids in response to WW and freezing with little impact on the unsaturated fatty acids. Despite these species differences, the changes in fatty acids reported here coincided with the imposed freezing stress as also shown for other plants (Uemura and Steponkus 1999; Dalmannsdóttir et al. 2001; Strimbeck et al. 2015). Overall, these results suggest that the success of the grass species in this study may in part be due to their capability to modify the fatty acid composition of their tissues. However, other physiological aspects, such as metabolites, protein and cell wall composition, which were not measured, could have also played a role (Hughes and Dunn 1996). Other adaptive mechanisms enhancing winter survival of grasses are the meristem placement at the base of the plant, where temperature fluctuations are damped and the growth of new leaves from the meristem in the next growing season (Bell 1974; Barton 2010). It is possible that other deciduous plants that have previously been shown to be resistant to field simulations and natural extreme winter warming events (Bokhorst et al. 2011, 2015) also have similar physiological adaptations and strategies.

Quantification of *CBF* expression was unfortunately unsuccessful for grasses, and therefore we cannot make comparisons between the different plant groups in this study. *CBF*s are DNA-binding transcriptional activator proteins, which regulate pathways affecting cold acclimatization and freezing tolerance. Higher *CBF*-protein levels in *Arabidopsis thaliana* increases frost tolerance and silencing of *CBF* can lead to decreased frost tolerance (Jaglo-Ottosen et al. 1998; Oakenfull et al. 2013). The strong increases in *CBF* expression following − 10 and − 20 °C freezing in *Empetrum* and *Vaccinium* can be assumed to be associated with frost resistance (Jaglo-Ottosen et al. 1998). However, as *CBF* expression occurred after freezing, it did not protect against damage and mortality during freezing for these dwarf shrubs. Adaptations by plants to the rapid temperature changes of extreme winter warming events may be problematic, if high *CBF* expression is required during freezing events. These aspects need to be explored in further detail if we want to identify the genetic aspects of sub-Arctic plant responses to winter temperature stress under climate change.

A third objective of this study was to identify which winter abiotic factor was responsible for the plant damage and mortality following extreme winter warming events. Freezing to − 20 °C resulted in biomass declines across most study species, but it did not consistently increase the damage following the winter warming events. Instead, some species suffered complete dieback following the winter warming treatment (*Pinus* and *Picea*), while others were more sensitive to the freezing treatments (*Vaccinium* and *Empetrum*). *Poa* and *Phleum* showed increased mortality following WW with freezing. These results do not provide direct insight into the physiological mechanism, such as ice crystallization, supercooling and frost injury (Beck et al. 2004; Liu and Osborne 2008), behind the observed plant mortality and growth reductions, but can be used to predict plant community assembly when taking winter freezing and snow conditions into account. The loss of snow cover did reduce growth for many species, and growth reductions were greatest in plants (*Festuca*, *Poa*, *Phleum* and *Pyrola*) not receiving a new snow cover after the winter warming event. Snow cover loss exposed plants to increased temperature variability but also to winter sunlight, which eventually would lead to evaporation losses that influence winter physiological processes and cause greater stress (Heide 1993; Neuner et al. 1999; Adams et al. 2002; Starr and Oberbauer 2003; Siffel and Santrucek 2005). Although we cannot identify the specific mechanism and causes behind the observed changes in biomass following shallower snow regimes, this aspect of the winter ecology obviously plays an important role in plant growth (Bokhorst et al. 2016). Therefore, the drivers behind plant vulnerability to winter temperature extremes and changes in snow cover appears to be highly species specific, but the responses observed here can be used to predict how local plant communities may respond to future Arctic climate scenarios. As such, we can expect that evergreen plants will be negatively impacted following increased winter temperature and snow variability, while grasses, especially *Poa* and *Festuca*, and deciduous plants are better adapted to these potentially stressful conditions (Kreyling et al. 2008; Bokhorst et al. 2015).

Finally, we expected that higher N availability would promote plant growth, but simultaneously increased vulnerability to freezing stress due to changes in cell and physiological characteristics associated with drought and frost susceptibility (Macgillivray et al. 1995; Carroll et al. 1999; Schaberg et al. 2002). The grasses and *Betula* responded positively to N additions, while the dwarf shrubs and evergreen seedlings did not. These results most likely reflect the faster growth rate of the deciduous species over the evergreen species (Cornelissen et al. 1996; Wright et al. 2004). Multiple growing seasons with higher N availability may also result in increased growth of the evergreen study species (Aerts 2010). The higher N availability did interact with WW and freezing treatments leading to higher membrane damage and mortality of *Poa*. However, the majority of species did not show any response to the interaction of N and temperature variability. Therefore, higher N availability does not necessarily increase vulnerability to freezing stress (Taulavuori et al. 2014). However, the N loading in this experiment was chosen at a relatively low level (Phoenix et al. 2012) to simulate realistic pollution levels, and loadings beyond natural levels may induce higher vulnerability to the combination of stressors.

In conclusion, the evergreen dwarf shrubs and tree seedlings were more susceptible to extreme winter warming events than grasses and *Betula*. Physiological activity during the extreme winter warming event was not a precursor for growth reduction and mortality rates. Instead, plant species that changed their membrane fatty acid composition in response to the winter warming and freezing showed least growth reductions and mortality. Furthermore, the drivers (winter warming event or freezing) of growth reductions and mortality were not consistent between species and functional groups, making it hard to make predictions for large plant group responses to climate scenarios. However, this work does indicate that evergreen plants appear the most vulnerable, compared to grasses and other deciduous plants, when facing future warmer winter climate conditions.

### *Author contribution statement*

SB and JWB designed and conducted the experimental work. All authors wrote or commented on the main text and supplementary notes. LJ and KK were responsible for the *CBF* gene analyses, and GKE and HKM were responsible for the fatty acid analyses.

## Electronic supplementary material

Below is the link to the electronic supplementary material.

Supplementary material 1 (DOCX 1523 kb)

## Abbreviations

C: Control
CBF: C-repeat binding factor
N: Nitrogen
S: Snow
TC: Treatment control
WW: Extreme winter warming
