# Supplementary material for: Contrasting survival and physiological responses of sub-Arctic plant types to extreme winter warming and nitrogen
Source: Planta. 2017 Nov 21;247(3):635–48. doi: 10.1007/s00425-017-2813-6 (PMC5809542; doi:10.1007/s00425-017-2813-6)
Supplement: Supplementary file 1 — Supplementary material 1 (DOCX 1523 kb) [file 425_2017_2813_MOESM1_ESM.docx]

**Contrasting survival and physiological responses of sub-Arctic plant types to extreme winter warming and nitrogen**

Stef Bokhorst^*^, Laura Jaakola, Katja Karppinen, Guro K. Edvinsen, Hanne K. Mæhre, and Jarle W. Bjerke

* Department of Ecological Science, VU University Amsterdam, De Boelelaan 1085, 1081 HV Amsterdam, The Netherlands.

Email: s.f.bokhorst@vu.nl

**Table S1** Monthly temperature and sunlight hours for Tromsø during winter 2014. Data were obtained from the Norwegian Meteorological institute

|  | Temperature (°C) | |  | Sunlight hours |
| --- | --- | --- | --- | --- |
|  | Max. | Min. | mean | h/d |
| January | 3.8 | -13.0 | -5.6 | 00:03 |
| February | 6.9 | -9.8 | -0.1 | 01:31 |
| March | 5.7 | -10.6 | -0.6 | 03:13 |
| April | 9.4 | -6.7 | 1.1 | 05:42 |

**Table S2** Gene-specific primers used for quantitative reverse transcription PCR (qRT-PCR) analyses

| Gene | Primer sequence 5’-3’ |
| --- | --- |
| *Empetrum CBF* | CCTGAAAGGCGATTCTTCGTTG (forward) |
|  | TCATCAATCCTCGAACCACCAG (reverse) |
| *Vaccinium CBF* | GGAGGAGGAACAACGACAGG (forward) |
|  | GCGAAATTCAAACAGGCCGT (reverse) |
| *Empetrum 18S rRNA* | TTCCTAGTAAGCGCGAGTCATC (forward) |
|  | CCTTGTTACGACTTCTCCTTCC (reverse) |
| *Vaccinium 18S rRNA* | CGCTGGCACCTTATGAGAAATC (forward) |
|  | ATGCACCACCACCCATAGAATC (reverse) |

**Supporting information Fig. S1** Graphical presentation of the experimental workflow. Nine plant species were planted in pots (*n* = 280 for each species) of the experimental garden in Holt during summer 2013 (see materials and methods). These plants were removed from the field during January 2014 and subsequently exposed to simulations of extreme winter warming events (WW: 6.0 °C) or a treatment control (TC: 0.5 °C) and freezing temperatures under controlled conditions in dark climate chambers after which the plants were returned to the experimental garden for the remainder of winter. In addition, there was a nitrogen addition (+N and –N) treatment added during summer 2013 and a snow removal (+S and –S) treatment following the climate room treatment in January 2014

**
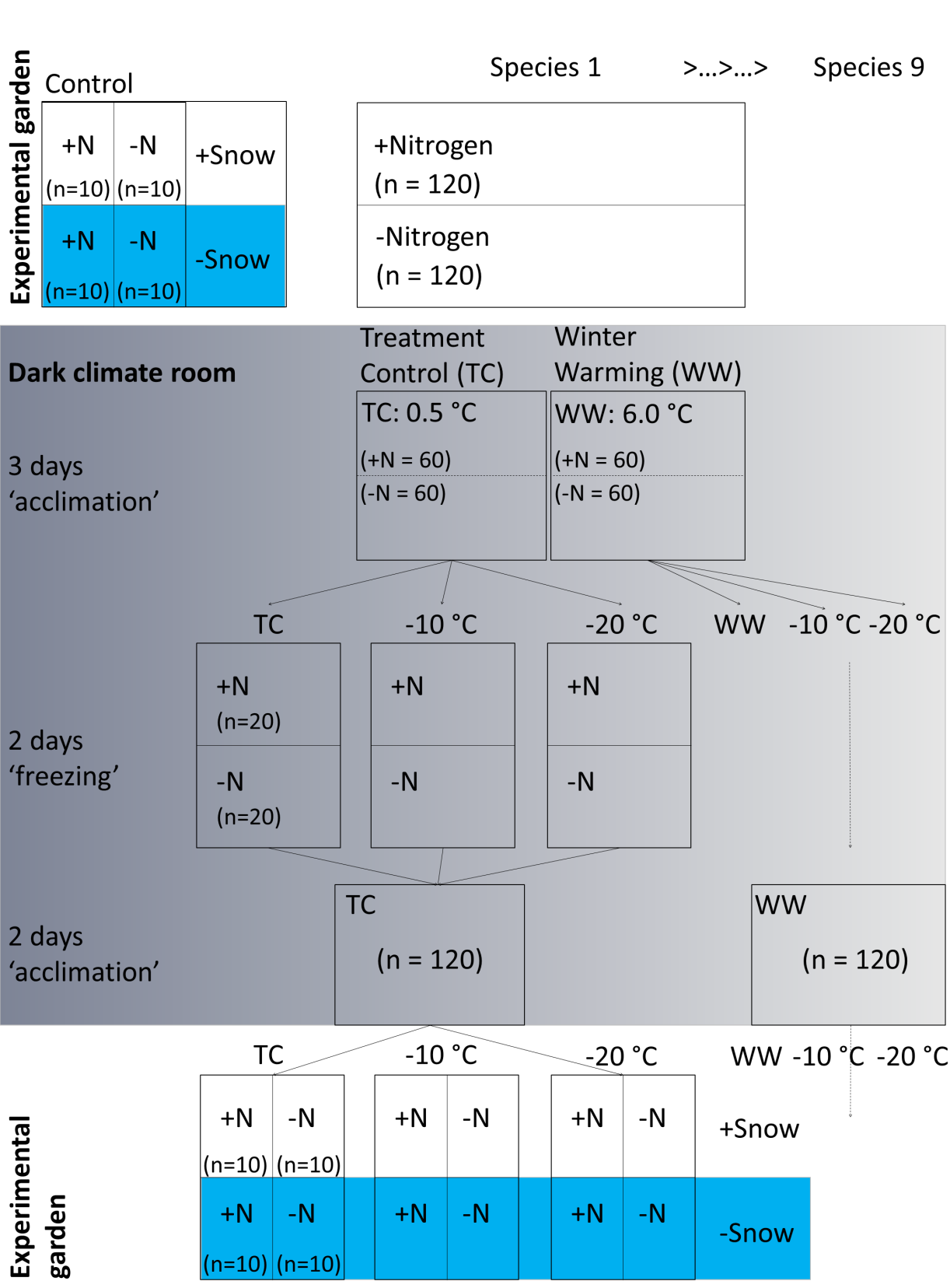
**

**Supporting information Fig. S2** Potential activity of photosystem II (Fv/Fm) of deciduous (grasses) and evergreen plants exposed to different winter temperature regimes. The treatments included a treatment control (TC at 0.5 °C), a winter warming treatment (WW at 6 °C) and freezing exposure to -10 °C and -20 °C. Bars with different letters are significantly different from one another (Tukey HSD P < 0.05). Bars are mean of *n* = 20 and error bars are 1 SE





**Supporting information Fig. S3** Electrolyte leakage of leaf tissue of deciduous (birch and grasses) and evergreen plants exposed to different winter temperature regimes. The treatments included a treatment control (TC at 0.5 °C), a winter warming treatment (WW at 6 °C) and freezing exposure to -10 °C and -20 °C. Bars with different letters are significantly different from one another (Tukey HSD *P* < 0.05). Bars are means of *n* between 9 and 30 replicates, as sample sizes varied across species. Error bars are ±1 SE





**Supporting information Fig. S4** Principal Component bi-plot of the fatty acid membrane composition of various sub-Arctic plants exposed to different winter temperature regimes (indicated by different symbols and colours). The treatments included a treatment control (TC at 0.5 °C), a winter warming treatment (WW at 6 °C) and freezing exposure to -10 °C and -20 °C. Grey triangles represent the membrane fatty acids. The percentage of variance of the data explained by PC1 and PC2 are shown along the x and y axes

**

**

**Supporting information Fig. S5** Expression of *CBF* genes in response to extreme winter warming temperatures for *Empetrum* (**a**) and *Vaccinium* (**b**). The treatments included a treatment control (TC at 0.5 °C), a winter warming treatment (WW at 6 °C) and freezing exposure to -10 °C and -20 °C. Samples were also collected following the freezing exposure. Bars are mean of *n* = 2-9 replicates as sample size varies across species and treatments. Error bars are 1 standard deviation (SD)

**

**
